# Paragangliome mésentérique

**DOI:** 10.11604/pamj.2017.26.142.11813

**Published:** 2017-03-14

**Authors:** Hanan El Ouahabi, Nadia Anoun

**Affiliations:** 1 Service de Diabétologie, Endocrinologie CHU Hassan II Fès, Faculté de Médecine, Fès, Maroc

**Keywords:** Tumeur mésentérique, paragangliome, traitement chirurgical, Mesenteric tumor, Extra-adrenal paraganglioma, surgical management

## Image en médecine

Les paragangliomes extra-surrénaliens surviennent rarement de façon aberrante en dehors de la région parallèle au système nerveux autonome s'étendant de la région cervicale à la région pelvienne. Nous rapportons un cas rare de paragangliome localisé au niveau du mésentère chez une femme de 58 ans sans antécédents pathologiques notables, opérée quatre mois avant son admission pour une volumineuse masse abdominale mesurant 14 x 12 cm, découverte sur un examen tomodensitométrique, réalisé devant des douleurs abdominales diffuses. Le résultat anatomopathologique complété d'une étude immuno-histochimique a révélé un paragangliome de localisation mésentérique, pour lequel elle nous a été adressée pour complément de prise en charge. La patiente était en bon état général, normo tendue, asymptomatique. L'examen abdominal notait une cicatrice de laparotomie médiane à cheval sur l'ombilic. Elle avait bénéficié d'un dosage des dérivés méthoxylés urinaires de 24h revenus normaux. Un bilan d'extension fait d'une TDM thoraco-abdomino-pelvienne avait révélé une thrombose partielle du tronc porte et du tronc spléno mesaraïque et de la veine cave inférieure sans autre localisation secondaire. La patiente fut mise sous traitement anticoagulant à dose curative.

**Figure 1:**
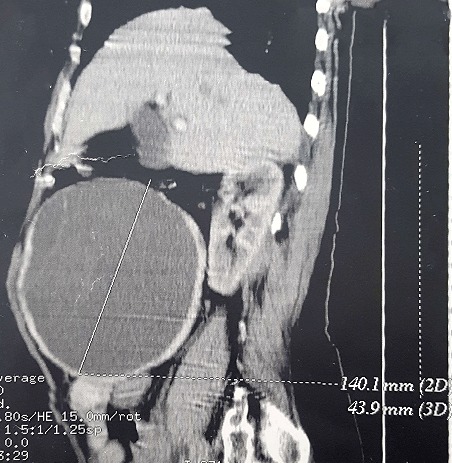
TDM abdominale en coupe sagittale montrant la tumeur

